# Planning for a publicity campaign on use of antibiotics and antibiotic resistance in Hong Kong

**DOI:** 10.1186/1753-6561-5-S6-O45

**Published:** 2011-06-29

**Authors:** ML Ho, MK Tham, WH Seto, TS Lam, LC Wong, TY Wong

**Affiliations:** 1 Centre for Health Protection, Hong Kong, China; 2 Hospital Authority, Hong Kong, China

## Introduction / objectives

Hong Kong presents the first published community-wide publicity campaign on use of antibiotics and antibiotic resistance being conducted in Asia.

## Methods

Liaison with stakeholders started early. Literature and local data were reviewed. A population-based telephone survey was commissioned to measure knowledge, attitude, practice of the public related to antibiotics and their awareness of antibiotic resistance. Random sample was drawn from the latest residential telephone directory. A structured questionnaire was used for adults 18 years or above. Baseline survey was conducted in November 2010 and results were used to formulate health messages for the campaign in March/April 2011. Follow-up survey would be conducted in June/July 2011.

## Results

1569 respondents were successfully interviewed in baseline survey. Response rate was 69%. 34% and 67% believed antibiotics could cure flu and viral infections respectively. Misunderstanding was especially noted in female, older, married, lower education level or household income. 56% heard of antibiotic resistance. Television was the most common information source. They gave a higher rating of impact to information obtained from health professionals. A simple message, “antibiotics do not help in cold and flu”, was thus adopted and the public was encouraged to ask their doctors about medications prescribed. Besides printed materials, announcement of public interest was developed for television and radio. Resources were uploaded to Centre for Health Protection website. Doctors and pharmacists were informed of the survey results to enlist their support to the campaign.

## Conclusion

A baseline survey helped formulation of key messages and provided evidence to convince stakeholders.

## Disclosure of interest

None declared.

